# Effectiveness of an *Arthrospira platensis* (Spirulina) Softgel Supplementation on Sleep Quality, Mental Health Status, and Body Mass Index in Mild to Moderately Severe Depression Adults: A Double‐Blinded, Randomized, Placebo‐Controlled Trial

**DOI:** 10.1002/fsn3.70082

**Published:** 2025-03-05

**Authors:** Phenphop Phansuea, Kittipat Chotchindakun, Yuraporn Sahasakul, Karunpong Phattaramarut, Panwong Kuntanawat

**Affiliations:** ^1^ Food and Nutrition Academic and Research Cluster, Institute of Nutrition Mahidol University Nakhon Pathom Thailand; ^2^ Institute of Research and Development Suranaree University of Technology Nakhon Ratchasima Thailand; ^3^ Biomedical Engineering Institute Chiang Mai University Chiang Mai Thailand; ^4^ Biomedical Engineering and Innovation Research Center Chiang Mai University Chiang Mai Thailand; ^5^ Office of Research Administration Chiang Mai University Chiang Mai Thailand; ^6^ School of Psychiatry, Institute of Medicine Suranaree University of Technology Nakhon Ratchasima Thailand; ^7^ School of Biotechnology, Institute of Agricultural Technology Suranaree University of Technology Nakhon Ratchasima Thailand

**Keywords:** *Arthrospira platensis*, mental health, RCT, sleep quality, spirulina

## Abstract

*Arthrospira platensis* (Spirulina) has been recognized for its diverse benefits to human health. Currently, stress and sleep disorders are prevalent and increasing annually worldwide. This study aimed to conduct a randomized controlled trial to assess the effects of Spirulina on sleep quality, mental health status, and body mass index (BMI) measurements in adults with mild to moderately severe depression. Sixty‐six participants with mild to moderately severe depression were randomly assigned to receive either 2 g/day of Spirulina (two 1000 mg capsules) (*n* = 33) or a placebo (*n* = 33) for 8 weeks. Assessments conducted at baseline, week 4, and the end of the intervention included General Interview Questionnaires (GIQ), BMI, pulse and blood pressure measurements, the Depression Anxiety Stress Scales (DASS‐21), the Pittsburgh Sleep Quality Index (PSQI), and the Sleep Hygiene Index (SHI). Sixty‐three participants completed the trial. Adherence to supplementation was high, with two participants in the Spirulina group reporting missed doses for one day and 3 days, respectively. Out of 66 participants, three from the control group dropped out by the end of the study. Analysis using repeated measures ANOVA revealed a significant difference in PSQI scores in the experimental group from baseline to 8 weeks, with the mean score decreasing from 7.03 ± 3.52 to 4.97 ± 1.98. The control group's mean score decreased from baseline to week 4, followed by a slight increase by week 8. Comparing the experimental and control groups at week 8, the mean PSQI scores differed significantly, with 4.97 ± 1.98 and 6.73 ± 2.69 (95% CI [−2.945, −0.582], *p* = 0.004). Significant differences were found in sleep quality and sleep latency between the groups, with scores of 0.82 ± 0.58 and 1.33 ± 0.61 (95% CI [−0.815, −0.215], *p* = 0.001) and 0.79 ± 0.70 and 1.33 ± 0.71 (95% CI [−0.900, −0.191], *p* = 0.003), respectively. Both groups showed statistically significant decreases in DASS‐21 scores. The experimental group exhibited gradual reductions across all assessments (19.27 ± 13.09, 14.24 ± 10.13, 11.58 ± 8.19) at *p* < 0.05. Conversely, the control group saw an increase in mean scores by week 8 (15.43 ± 9.18, 12.57 ± 9.83, 12.63 ± 10.50). BMI indices and blood pressure exhibited no statistically significant changes (*p* > 0.05). Sleep quality, sleep latency, and mental health improved after *Arthrospira platensis* daily softgel supplementation. Further clinical studies, potentially conducted over extended periods, could provide additional support for the trends observed in this study.

## Graphical abstract

*Arthrospira platensis* (Spirulina) has been recognized for its diverse benefits to human health. Currently, stress and sleep disorders are prevalent and increasing annually worldwide. Sleep quality, sleep latency, and mental health improved after daily supplementation of *Arthrospira platensis*.

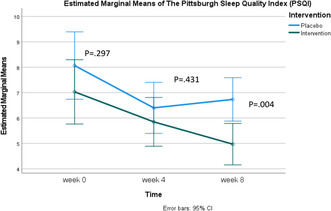

## 1 Introduction

The mental well‐being of the population constitutes a fundamental pillar for a robust society and economy (Dich et al. 2019). However, following the widespread outbreak of COVID‐19 in 2019, mental health issues have become a subject of considerable concern. Previous research consistently indicates that the global populace has been subjected to heightened levels of stress compared to pre‐pandemic times. This includes fear of infection, grief from losing loved ones, abrupt lifestyle alterations, physical distancing, and various challenges, such as economic crises, with accompanying socio‐psychological ramifications (Ruengorn et al. 2022). These factors have led to accumulative stress, sleep disturbances, and fatigue and may potentially culminate in depressive disorders (Phu et al. 2023). These adverse consequences affect not only the patients but also their caregivers, siblings, and romantic partners, and contribute to a reduction in the overall quality of the population, thereby impeding national developmental progress (Prasartpornsirichoke et al. 2023).

Mild to moderately severe depression in adults presents a substantial public health burden, affecting millions globally and often leading to profound individual and societal consequences if left untreated (Park and Zarate Jr. 2019). Individuals with this level of depression experience significant impairments in daily functioning, including diminished productivity, strained relationships, and decreased quality of life. The economic impact is considerable, with increased healthcare costs, reduced workforce participation, and higher disability rates. Previous studies in Thailand have also reported a continuous increase in the prevalence of this population group (Sahasakul et al. 2023; Pudpong et al. 2021). Furthermore, untreated depression can escalate, potentially leading to severe mental health crises, including suicidal ideation and behavior.

Sleep problems represent a prevalent health issue, affecting approximately 10 to 20% of the global population, with a higher incidence observed in females, the elderly, and individuals with mental health concerns (Morin and Jarrin 2022). In Thailand, according to the report from the Department of Mental Health, Ministry of Public Health, in the year 2020, it was found that there were over 19 million individuals experiencing insomnia, and this trend continues to rise steadily due to socio‐environmental and health‐related factors. Insomnia can be treated or alleviated by administering synthetic sleep‐inducing agents, such as benzodiazepines, zopiclone, and zolpidem, which stimulate or inhibit sleep‐related neurotransmitters, leading to episodic sleep (Lie et al. 2015). While these pharmacological agents effectively mitigate insomnia, their transient mechanism of action necessitates their consideration as palliative measures. Furthermore, their use may give rise to undesirable effects in both short‐term and long‐term contexts, such as respiratory suppression, memory impairments, drug tolerance, and dependency (Rogers et al. 2023). Therefore, addressing insomnia through regulating neurotransmitter levels related to sleep and maintaining them within appropriate bounds in accordance with natural physiological rhythms represents a fundamental and efficacious approach to rectifying the root cause of sleep disturbances.

Based on data from individuals experiencing insomnia, there is an evident trend of diminished levels of crucial neurotransmitters associated with sleep in the serotonin (5‐hydroxytryptamine; 5‐HT) group, which is linked to reduced quantities of tryptophan, a precursor substance for 5‐HT synthesis (Owens 2014). Consequently, rectifying insomnia by utilizing food ingredients with elevated levels of tryptophan represents an effective and sustainable approach to addressing the root cause of sleep disturbances (Sutanto et al. 2022). One noteworthy food ingredient is *Arthrospira platensis* (Spirulina), which is generally recognized as safe (GRAS) for consumption in both food and supplement forms over an extended period and contains approximately 900–1000 mg of tryptophan per 100 g of weight (Liestianty et al. 2019). In a study involving chronic ulcerative colitis patients receiving Spirulina (1000 mg/day), statistically significant reductions in sleep disturbances and stress scores were observed (*p* < 0.05) compared to the placebo group. Additionally, the quality of life for ulcerative colitis patients significantly improved (*p* = 0.03) (Mohammadi et al. 2019). Aligning with findings in experimental mice, Spirulina was found to substantially lower levels of stress and anxiety (*p* < 0.05) and exhibited antidepressant properties (Moradi‐Kor et al. 2020). Furthermore, Spirulina serves as a vital source of various compounds, including carotenoids, phycocyanin, vitamin B1, and gamma‐linoleic acid, all of which possess diverse pharmacological properties such as antioxidant, anti‐inflammatory, and immunostimulatory effects, contributing to an integrated approach to healthcare, and likely to stress control and sleep health, with established safety records and historical human consumption (Finamore et al. 2017).

To date, there has been no clinically published study demonstrating the sleep quality and mental health effects of *Arthrospira platensis* (Spirulina) supplementation in the mild to moderately severe depression adult population. Therefore, this study aimed to investigate the effectiveness of *Arthrospira platensis* (Spirulina) supplementation in the form of softgel capsules on sleep quality and mental health status among this population.

## 2 Materials and Methods

### 2.1 Population and Sample

Participants were recruited by distributing posters within the proximity of Mahidol University, Salaya campus, Nakhon Pathom province, Thailand.

The study included individuals aged 18–60 years in robust health without systemic ailments, with a depression score (PHQ‐9) of 5–19 points and a body mass index (BMI) not exceeding 30 kg/m^2^. Exclusion criteria encompass pregnant or breastfeeding individuals within the past 3 months, night shift workers or those with continuous 24‐h schedules, severe psychiatric conditions requiring medical intervention, recent use of weight‐loss medications, consumption of sleep‐inducing supplements or melatonin‐containing products within the past 3 months, smoking more than 10 cigarettes daily, regular alcohol consumption exceeding 14 drinks per week for males or 7 drinks per week for females, recent international travel or time zone changes, and allergies to spironolactone or a sugar‐free syrup derived from Luo Han Guo.

### 2.2 Study Design and Ethics Approval

The trial followed the CONSORT statement (Cuschieri 2019); a randomized, placebo‐controlled, parallel‐arm trial was conducted at the Institute of Nutrition, Mahidol University (INMU). The present investigation employed a straightforward randomization technique utilizing a random number table. This method ensured that researchers and participants remained unaware of the supplement allocation until the study's conclusion. Additionally, at the commencement of the supplementation phase, softgel capsules were dispensed in packages labeled 01 to 66, thereby effectively blinding both the researcher and participants. Participants who met the inclusion criteria and were willing to participate were enrolled in a randomized controlled trial (1:1). General information, mental health status, sleep quality, and sleep hygiene were evaluated using a general information questionnaire (GIQ), Depression Anxiety Stress Scales (DASS‐21) (Wittayapun et al. 2023), Patient Health Questionnaire (PHQ‐9) (Lotrakul et al. 2008), Thai Version of the Pittsburgh Sleep Quality Index (Thai‐PSQI) (Sitasuwan et al. 2014), and Sleep hygiene index (SHI) (Kerdcharoen et al. 2020) were conducted at baseline, week 4, and week 8, respectively. Furthermore, we asked the participants to continue eating their usual diet and activities. All participants received unlabeled sachets of the assigned product so that they were blinded from their group identity.

This study received approval for the trial from the Mahidol University Central Institutional Review Board (MU‐CIRB) (Protocol Number: MU‐CIRB 2023/314.0610) for research involving the use of the trial (COA No. MU‐CIRB 2023/184.1312; Date of Approval: December 13, 2023). The data supporting the findings of this study are available from the corresponding author upon reasonable request.

### 2.3 Sample Size Calculation

The G*Power version 3.1.9.4 software (Kang 2021) was employed to conduct a power analysis, determining that a total of 26 participants was required to detect statistically significant trial effects, with a probability error of 0.05, a statistical power of 80%, and an effect size of 0.8 (Moradi‐Kor et al. 2020). Anticipating a potential dropout rate of 20%, the definitive sample size was established at 33 participants within each respective group, culminating in an aggregate sample size of 66 participants (Figure 1).

### 2.4 Intervention and Materials

**FIGURE 1:**
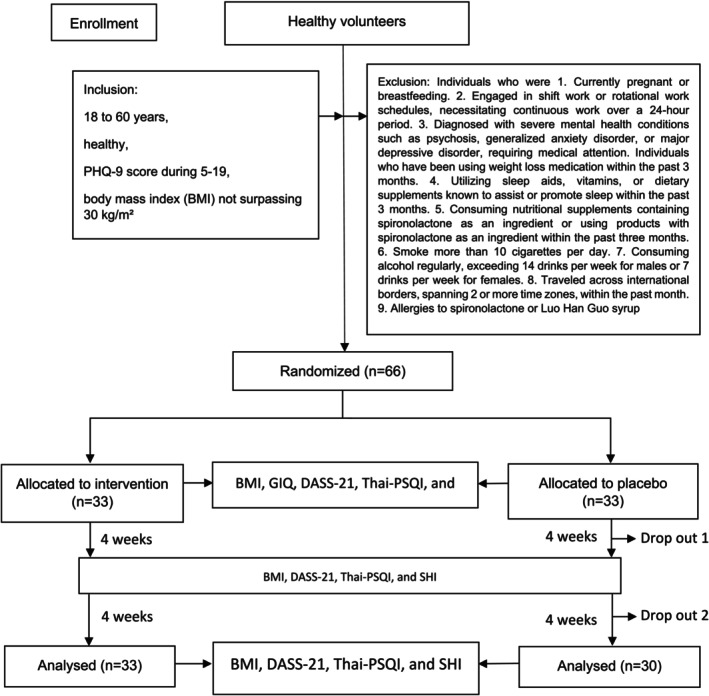
Study consort.

### 2.5 Research Procedure

The research team initiated the study by disseminating invitation posters in the vicinity of the INMU, both physically and online. Individuals expressing interest in participating were administered a screening questionnaire consisting of 21 questions, comprising 12 general information inquiries and 9 items from the Patient Health Questionnaire (PHQ‐9) for depression assessment. This process was anticipated to take approximately 3–5 min. After successful screening and study acceptance, participants were contacted to schedule appointments, specifying the date, time, and location. Additionally, the following instructions were provided: (1) For dietary and beverage intake, participants should fast or wait approximately 2–3 h after a meal before measurements. Up to two glasses of water may be consumed up to 2 h prior to assessment. Avoiding caffeinated beverages and diuretics is required for a minimum of 4 h before measurement, and abstaining from alcoholic beverages is necessary for 24 h beforehand. (2) Regarding other preparatory measures, participants should wear the lightest possible attire and remove any jewelry or accessories with substantial weight. If experiencing urinary discomfort, participants should void approximately 30 min before assessment. After intense physical activity, sauna use, or activities resulting in significant perspiration, assessments should be conducted after approximately 3 h or before engaging in such activities. Consistency in the timing of previous assessments and food intake and activity levels is essential for effectively evaluating changes in physiological components.

### 2.6 Intervention

**FIGURE 2:**
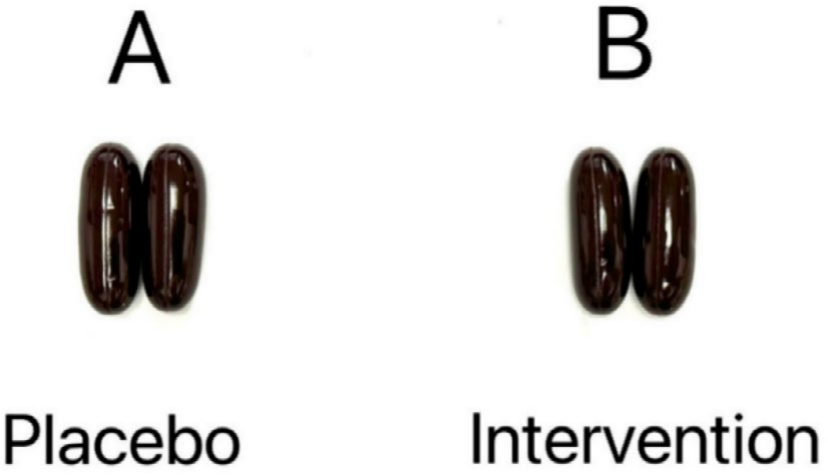
Placebo and intervention capsules.

*Arthrospira platensis* (Spirulina), a cyanobacterium characterized by greenish‐blue coloration, is a consumable microalga. The Spirulina (Sup Blue; SUP‐B) utilized in this study was initially identified by selecting wild specimens from Nakhon Ratchasima Province, Thailand. The intervention in this study involved the administration of SUP‐B at a dosage of 2 g in softgel form, equivalent to 2 softgel capsules (1 g per softgel capsule) per day. Softgel capsules are superior to traditional capsules as they offer better bioavailability, ensuring more efficient absorption of the active ingredients by the body. Additionally, softgels are easier to swallow and do not have the unpleasant taste or odor often associated with traditional capsules. This was accompanied by a placebo product, a sugar‐free syrup derived from Luo Han Guo, also administered at 2 g in the same softgel capsule format and dosage. Both interventions were provided by Siambiota Co. Ltd., a manufacturer adhering to Good Manufacturing Practices (GMP), with particular emphasis on the production standards of SUP‐B. The intervention period lasted for 8 weeks (Figure 2).

### 2.7 Data Acquisition

#### 2.7.1 General Interview Questionnaires (GIQ)

GIQ of Personal Factors of Research Participants comprises a set of 14 questions encompassing the following aspects: gender, age, highest educational attainment, occupation, monthly income, history of consumption of seaweed or freshwater algae, and exercise history.

#### 2.7.2 Body Mass Index (BMI)

BMI was measured by INBODY 270 (McLester et al. 2020). The body composition was conducted at baseline, week 4, and at the end of the study.

#### 2.7.3 Pulse and Blood Pressure

Pulse and blood pressure were measured by Omron HEM‐1040 (Takahashi and Saito 2020), and participants received guidance and instructions for measurement and recording from trained researchers. Pulse and blood pressure were assessed at baseline, week 4, and at the end of the study.

### 2.8 Mental Health Measurements

#### 2.8.1 Patient Health Questionnaire (PHQ‐9)

The Patient Health Questionnaire (PHQ‐9) is a self‐administered version of the PRIME‐MD diagnostic instrument for common mental disorders. It was developed in 1999 by Drs. Robert L. Spitzer and his colleagues. The overall accuracy of the questionnaire was 85%, sensitivity was 75%, and specificity was 90% (Spitzer et al. 1999). The internal reliability of the PHQ‐9 was at 0.89 in the PHQ Primary Care Study and 0.86 in the PHQ Ob‐Gyn Study (Kroenke et al. 2001). The PHQ‐9 is shorter than many of the other depression screening instruments and can be self‐administered. Responses are measured on a Likert scale with 4 levels, and the scoring criteria and interpretations of the total score are outlined as follows: scoring criteria: The severity of depression can be categorized based on total scores. A score of 1–4 indicates no depression, while a score of 5–9 signifies mild depression. Moderate depression is reflected in scores ranging from 10 to 14, and scores of 15–19 indicate moderately severe depression. Severe depression is characterized by scores between 20 and 27.

#### 2.8.2 Depression Anxiety Stress Scales (DASS‐21)

Depression Anxiety Stress Scales (DASS‐21) serve as a self‐assessment tool for negative emotional states encompassing three dimensions: depression, anxiety, and stress. It comprises a total of 21 items (Wittayapun et al. 2023). The instrument's reliability was assessed in terms of its Cronbach's alpha coefficients for depression, anxiety, and stress, yielding values of 0.28, 0.78, and 0.69, respectively. Each item was rated on a scale ranging from 0 to 3 points. The depression subscale comprised questions 3, 5, 10, 13, 16, 17, and 21; the anxiety subscale included questions 2, 4, 7, 9, 15, 19, and 20; and the stress subscale encompassed questions 1, 6, 8, 11, 12, 14, and 18. Responses are measured on a Likert scale with four levels, and the scoring criteria and interpretations are outlined as follows: scoring criteria: Symptoms of depression, anxiety, and stress can be classified into different levels based on their severity. Normal levels correspond to scores of 0–4 for depression, 0–3 for anxiety, and 0–7 for stress. Scores of 5–6 indicate mild symptoms of depression, 4–5 for anxiety, and 8–9 for stress. Moderate symptoms are characterized by scores of 7–10 for depression, 6–7 for anxiety, and 10–12 for stress. Severe symptoms are reflected by scores of 11–13 for depression, 8–9 for anxiety, and 13–16 for stress. Extremely severe symptoms are marked by scores of 14 or higher for depression, 10 or higher for anxiety, and 17 or higher for stress.

### 2.9 Sleep Quality Measurements

#### 2.9.1 The Pittsburgh Sleep Quality Index (PSQI)

The PSQI was developed in 1989 by researchers at the University of Pittsburgh (Buysse et al. 1989). It has been validated with a variety of clinical populations, including patients with major depressive disorder, disorders of initiating and maintaining sleep, cancer, and fibromyalgia. The questionnaire has an internal reliability coefficient (Cronbach's alpha) of 0.83, a test–retest reliability of 0.85 for the global scale, a sensitivity of 89.6%, and a specificity of 86.5% (Shahid et al. 2012).

PSQI is a self‐report questionnaire that assesses sleep quality over a 1‐month time interval. The questionnaire comprises 10 questions with 19 individual items. Each item belongs to one of seven subcategories: subjective sleep quality, sleep latency, sleep duration, habitual sleep efficiency, sleep disturbances, use of sleeping medication, and daytime dysfunction.

#### 2.9.2 Sleep Hygiene Index (SHI)

The SHI questionnaire (Mastin et al. 2006) possesses a Cronbach's alpha coefficient of 0.74. This index encompasses a total of 13 items and is a self‐reported measure designed by the International Classification of Sleep Disorders (ICSD) to evaluate sleep hygiene behaviors. Each item is rated on a Likert scale of five levels (“never,” “rarely,” “sometimes,” “often,” “always”). The total score ranges from 0 to 52, with higher scores indicating poorer sleep hygiene. The developers reported an internal consistency reliability coefficient (Cronbach's alpha) of 0.66 and a good test–retest reliability of 0.71 (Kerdcharoen et al. 2020; Mastin et al. 2006) from previous studies. A total score cut‐off of 16 points on the SHI demonstrated optimal sensitivity (77.0%) and specificity (47.5%) in identifying individuals classified as having poor sleep quality, as referenced against the PSQI (area under the curve = 0.65, 95% confidence interval = 0.59–0.71) (Seun‐Fadipe et al. 2018).

### 2.10 Statistical Analysis

Descriptive statistics were employed to analyze continuous variables for the baseline characteristics of participants. Measures of central tendency and dispersion were utilized to present continuous variables. Group categorical variables were presented using counts and percentages. All parameters were calculated at baseline, week 4, and week 8 and they were assessed using inferential statistics, independent *t*‐test (between groups), repeated measurement ANOVA (baseline, week 4, and week 8 within group), and Pearson Chi‐square test or McNemar's Chi‐square test (for categorical variable) as appropriate. Intention to treat and per protocol analyses were conducted after the trial finished. The significance of all statistical tests was evaluated at a significance level of 0.05. SPSS version 27.0 (IBM Corp., Armonk, NY, USA) was used for all statistical analyses.

## 3 Result

The study comprised 66 participants, with a mean age of 36.58 ± 11.70 years, of whom 72.7% were female. The average BMI was 22.91 ± 3.31 kg/m^2^. Regarding educational attainment, 84.9% of the participants had completed at least a bachelor's degree. The mean systolic blood pressure was 113.76 ± 13.96 mmHg, the diastolic blood pressure was 72.83 ± 9.82 mmHg, and the pulse rate was 76.12 ± 11.17 beats per minute. No significant differences were observed in the characteristics between the two groups (Table 1). Additionally, the results for BMI, systolic blood pressure, diastolic blood pressure, and heart rate at all three time points throughout the study showed no statistically significant differences (Supplementary B).

**TABLE 1:** Baseline characteristics between control and experimental groups (*n* = 66).

Characteristics	Total (*n* = 66)	Experimental (*n* = 33)	Control (*n* = 33)	*p*
Age (years)	36.58 ± 11.70	36.36 ± 11.76	36.79 ± 11.82	0.884
Gender				
Male	18 (27.3)	9 (27.3)	9 (27.3)	1.000
Female	48 (72.7)	24 (72.7)	24 (72.7)	
Educational level				
High school and lower	10 (15.1)	6 (18.1)	4 (12.1)	0.159
Bachelor's degree and upper	56 (84.9)	27 (81.9)	29 (87.9)	
Occupational				
University student	19 (28.8)	8 (24.2)	11 (33.3)	0.870
Private employees	22 (33.3)	12 (36.4)	10 (30.3)	
Government officer/State enterprise employees	17 (25.8)	9 (27.3)	8 (24.2)	
Freelance	8 (12.1)	4 (12.1)	4 (12.1)	
Income (THB)				
< 12,000	19 (28.8)	11 (33.3)	8 (24.2)	0.408
12,000–16,999	14 (21.2)	9 (27.3)	5 (15.2)	
17,000–20,999	8 (12.1)	2 (6.1)	6 (18.2)	
21,000–39,999	18 (27.3)	8 (24.2)	10 (30.3)	
≥ 40,000	7 (10.6)	3 (9.1)	4 (12.1)	
BMI (kg/m^2^)	22.91 ± 3.31	23.06 ± 3.25	22.76 ± 3.41	0.711
Underweight	6 (9.1)	3 (9.1)	3 (9.1)	0.697
Normal weight	28 (42.4)	12 (36.4)	16 (48.5)	
Overweight	11 (16.7)	7 (21.2)	4 (12.1)	
Obesity	21 (31.8)	11 (33.3)	10 (30.3)	
Systolic blood pressure (mmHg)	113.76 ± 13.96	111.73 ± 13.76	115.79 ± 14.07	0.240
Diastolic blood pressure (mmHg)	72.83 ± 9.82	71.18 ± 9.08	74.48 ± 10.39	0.174
Pulse rate (beats per minute)	76.12 ± 11.17	76.27 ± 10.57	75.97 ± 11.91	0.913

*Note:* Data expressed as a count (percentage) or the mean ± SD for categorical or continuous variables, respectively. Pearson's *χ* ^2^ test or independent *t*‐test was used to compare between groups for categorical or continuous variables, respectively. BMI classification: underweight (< 18.5), normal weight (18.5–22.9), overweight (23.0–24.9), obese (≥ 25.0).

Abbreviations: BMI, body mass index; mmHg, millimeters of mercury; kg/m^2^, kilogram per square meter; THB, Thai bht.

* Significant at the *p*‐value < 0.05.

Table 2 compares the mean PSQI scores between the experimental and control groups. At baseline and 4 weeks, the mean scores of both groups showed no significant differences, with values of 7.03 ± 3.52 and 8.07 ± 3.76 (95% CI [−2.723, 0.844], *p* = 0.298) and 5.85 ± 2.83 and 6.40 ± 2.67 (95% CI [−1.942, 0.839], *p* = 0.431), respectively. However, at 8 weeks, a statistically significant difference was observed between the experimental and control groups, with scores of 4.97 ± 1.98 and 6.73 ± 2.69 (95% CI [−2.945, −0.582], *p* = 0.004).

**TABLE 2:** Comparison of mean Pittsburgh Sleep Quality Index (PSQI) scores and subcomponents between control and experimental groups at baseline, week 4, and week 8 (Intention to Treat Analysis).

PSQI scores	Experimental (*n* = 33)	Control (*n* = 33)	*T*	95% CI	*p*
Baseline	7.03 ± 3.52^a^	8.07 ± 3.76^bc^	−1.052	−2.723, 0.844	0.297
Sleep quality	1.39 ± 0.75	1.48 ± 0.80	−0.478	−0.470, 0.289	0.634
Sleep latency	1.30 ± 1.08	1.64 ± 0.96	−1.327	−0.835, 0.168	0.189
Sleep duration	1.06 ± 0.86	1.12 ± 1.02	−0.260	−0.526, 0.405	0.796
Sleep efficiency	0.55 ± 0.75	0.91 ± 1.07	−1.595	−0.820, 0.093	0.116
Sleep disturbance	1.30 ± 0.59	1.24 ± 0.56	0.429	−0.221, 0.343	0.669
Sleep medication	0.30 ± 0.81	0.12 ± 0.33	1.194	−0.125, 0.489	0.239
Sleep dysfunction	1.30 ± 0.92	1.45 ± 1.06	−0.620	−0.640, 0.337	0.538
Week 4	5.85 ± 2.83	6.40 ± 2.67^b^	−0.793	−1.942, 0.839	0.431
Sleep quality	1.00 ± 0.66	1.20 ± 0.66	−1.196	−0.534, 0.134	0.236
Sleep latency	1.06 ± 0.79	1.20 ± 0.66	−0.755	−0.509, 0.230	0.453
Sleep duration	1.15 ± 0.94	0.87 ± 0.90	1.226	−0.180, 0.749	0.225
Sleep efficiency	0.42 ± 0.97	0.77 ± 0.97	−1.539	−0.787, 0.102	0.129
Sleep disturbance	1.21 ± 0.49	1.07 ± 0.37	1.353	−0.070, 0.361	0.181
Sleep medication	0.06 ± 0.24	0.13 ± 0.57	−0.668	−0.290, 0.145	0.506
Sleep dysfunction	0.94 ± 0.79	1.20 ± 0.89	−1.235	−0.683, 0.161	0.222
Week 8	4.97 ± 1.98^a^	6.73 ± 2.69^c^	−2.984	−2.945, −0.582	0.004*
Sleep quality	0.82 ± 0.58	1.33 ± 0.61	−3.434	−0.815, −0.215	0.001*
Sleep latency	0.79 ± 0.70	1.33 ± 0.71	−3.074	−0.900, −0.191	0.003*
Sleep duration	0.79 ± 0.65	1.07 ± 0.87	−1.451	−0.663, 0.105	0.152
Sleep efficiency	0.39 ± 0.61	0.53 ± 0.63	−0.893	−0.451, 0.173	0.375
Sleep disturbance	1.09 ± 0.46	1.07 ± 0.58	−0.184	−0.239, 0.287	0.854
Sleep medication	0.03 ± 0.17	0.13 ± 0.35	−1.471	−0.244, 0.038	0.149
Sleep dysfunction	1.06 ± 0.70	1.27 ± 0.87	−1.028	−0.608, 0.195	0.308

*Note:* Data expressed as a mean ± SD for continuous variables. Independent *t*‐test was used to compare between groups of continuous variables. Matching superscript pairs (aa,bb,cc) indicate statistically significant differences at the *p*‐value < 0.05 level, determined using repeated measures ANOVA.

Abbreviation: 95% CI, 95% confidence interval of the difference.

* Significant at the *p*‐value < 0.05.

**FIGURE 3:**
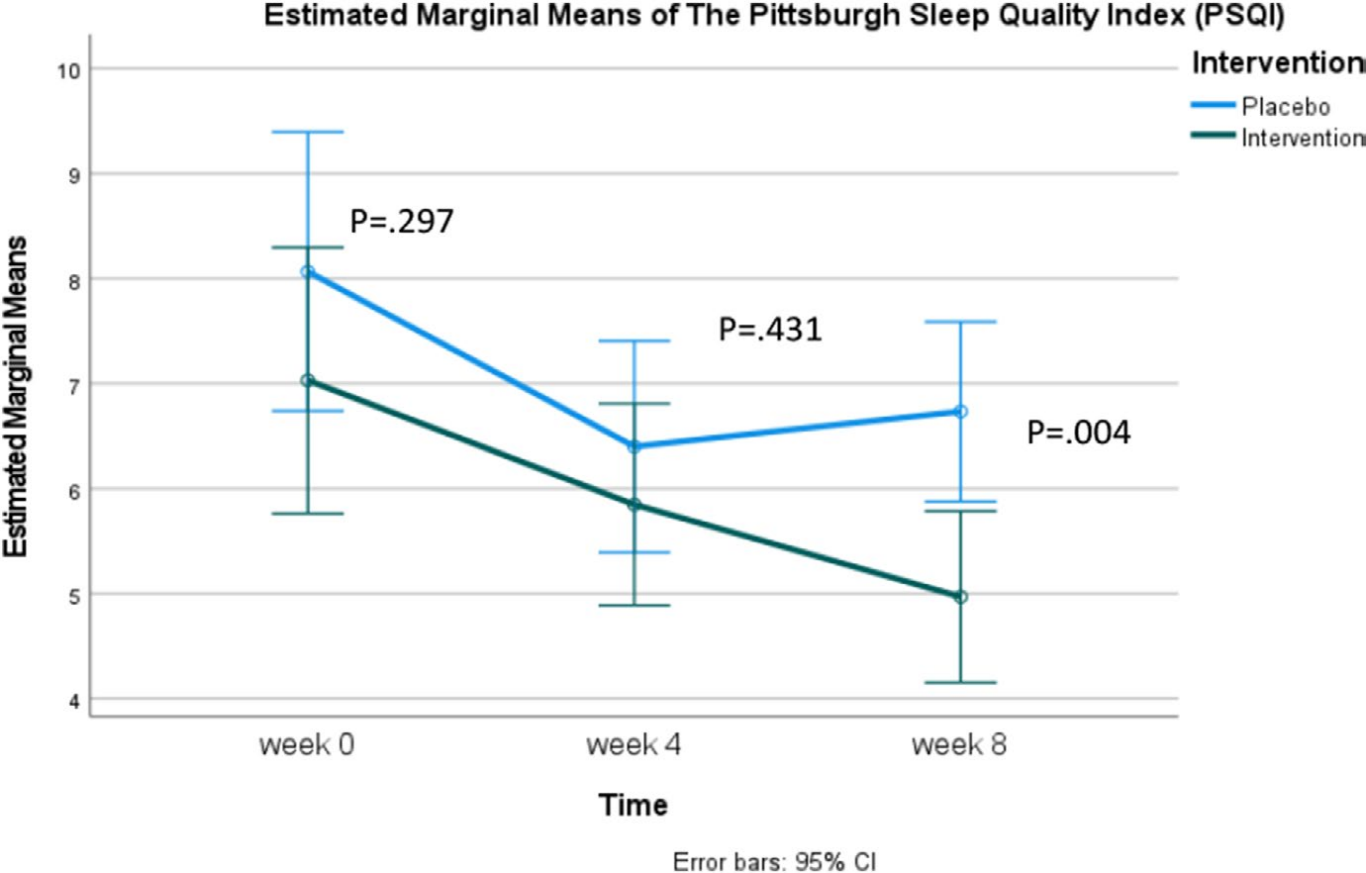
Estimated marginal means of the Pittsburgh Sleep Quality Index (PSQI).

Analysis using repeated measures ANOVA revealed a significant difference in the experimental group from baseline to 8 weeks, with the mean score decreasing from 7.03 ± 3.52 to 4.97 ± 1.98. In the control group, significant decreases were observed from baseline to weeks 4 and 8, with scores of 8.07 ± 3.76, 6.40 ± 2.67, and 6.73 ± 2.69, respectively. However, when considering Figure 3, it is evident that the control group's mean score decreased from baseline to week 4, followed by a slight increase by week 8.

**FIGURE 4:**
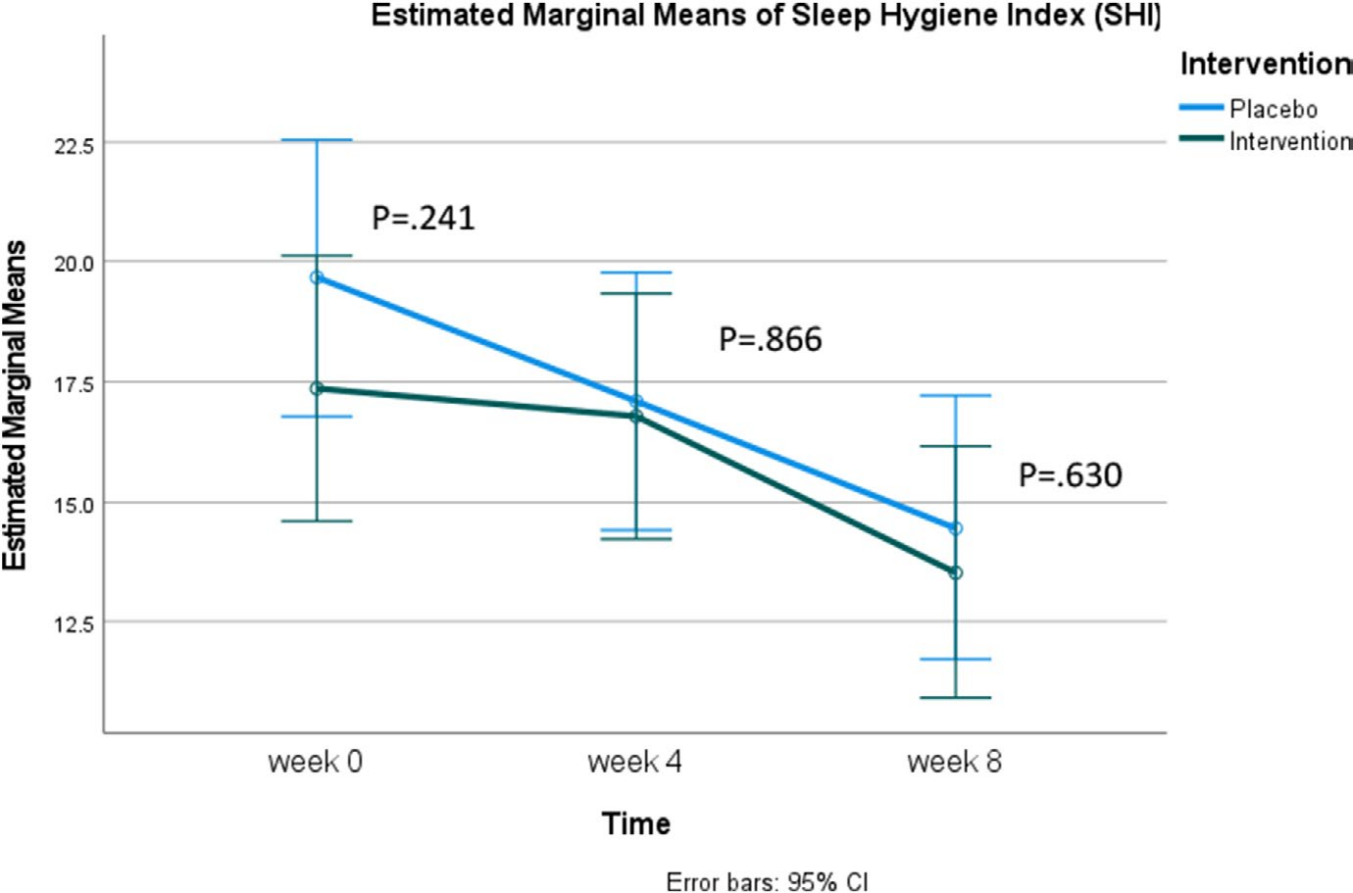
Estimated marginal means of Sleep Hygiene Index (SHI).

Table 3 compares mean SHI scores between the control and experimental groups at baseline, week 4, and week 8. The findings indicated no significant differences in sleep hygiene between the experimental and control groups across all time points. When examined over time, the experimental group showed significant decreases in mean sleep hygiene scores between baseline and week 8 (17.36 ± 7.13, 13.55 ± 6.92) and between week 4 and week 8 (16.79 ± 7.33, 13.55 ± 6.92). Similarly, the control group demonstrated significant differences in mean sleep hygiene scores between baseline and week 4 (19.70 ± 8.81, 17.10 ± 7.29), Baseline and Week 8 (19.70 ± 8.81, 14.47 ± 8.17), and week 4 and week 8 (17.10 ± 7.29, 14.47 ± 8.17). Considering Figure 4, it is evident that both groups exhibited trends of improved sleep hygiene over time (higher scores indicate poorer sleep hygiene).

**TABLE 3:** Comparison of mean Sleep Hygiene Index (SHI) scores between control and experimental groups at baseline, week 4, and week 8 (intention to treat analysis).

SHI scores	Experimental (*n* = 33)	Control (*n* = 33)	*T*	95% CI	*p*
Baseline	17.36 ± 7.13^a^	19.70 ± 8.81^cd^	−1.183	−6.275, 1.608	0.241
Week 4	16.79 ± 7.33^b^	17.10 ± 7.29^ce^	−0.0169	−4.000, 3.376	0.866
Week 8	13.55 ± 6.92^ab^	14.47 ± 8.17^de^	−0.0484	−4.723, 2.881	0.630

*Note:* Data expressed as a mean ± SD for continuous variables. Independent *t*‐test was used to compare between groups of continuous variables. Matching superscript pairs (aa,bb,cc,dd,ee) indicate statistically significant differences at the *p*‐value < 0.05 level, determined using repeated measures ANOVA.

Abbreviation: 95% CI, 95% confidence interval of the difference.

* Significant at the *p*‐value < 0.05.

**FIGURE 5:**
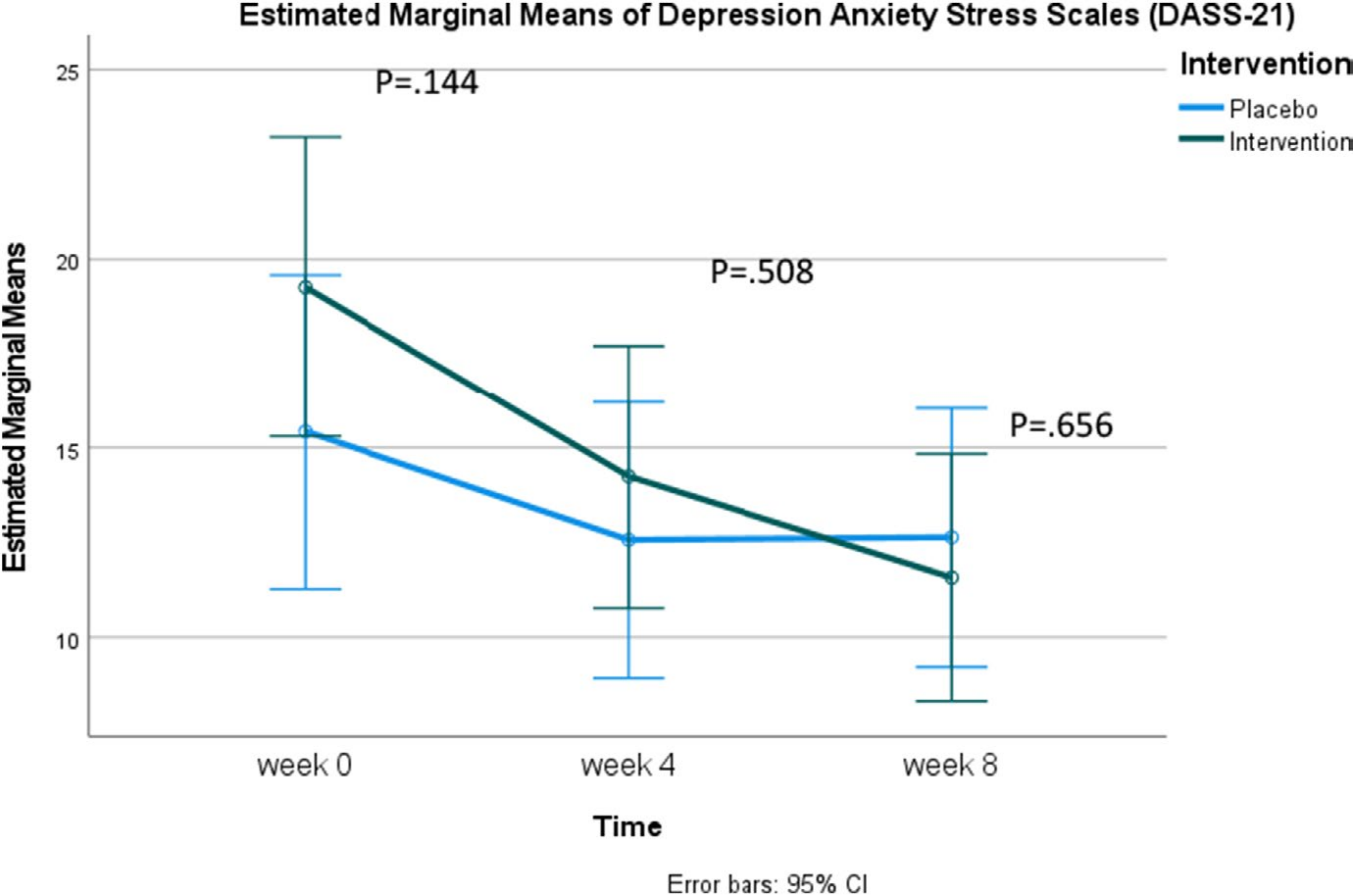
Estimated marginal means of Depression Anxiety Stress Scales (DASS‐21).

Regarding the mean scores of the Depression Anxiety Stress Scales (DASS‐21), there were no significant differences between the experimental and control groups at any single time point. However, when examined over time, the experimental group exhibited a statistically significant decrease in scores at each measurement interval: Baseline, week 4, and week 8 (19.27 ± 13.09, 14.24 ± 10.13, 11.58 ± 8.19, respectively). In the control group, there was also a statistically significant decrease in scores between baseline and week 4 (15.43 ± 9.18, 12.57 ± 9.83) and between baseline and week 8 (15.43 ± 9.18, 12.63 ± 10.50). Nevertheless, considering Figure 5, it is evident that while the experimental group's scores steadily decreased over time, the control group's scores slightly increased from week 4 to week 8 (Table 4).

**TABLE 4:** Comparison of mean Depression Anxiety Stress Scale (DASS‐21) scores and subcomponents between control and experimental groups at baseline, week 4, and week 8 (Intention to Treat Analysis).

DASS‐21 scores	Experimental (*n* = 33)	Control (*n* = 33)	*T*	95% CI	*p*
Baseline	19.27 ± 13.09^ab^	15.43 ± 9.18^de^	1.481	−1.441, 9.622	0.144
Depress	5.73 ± 5.09	4.39 ± 3.75	−1.212	−3.531, 0.865	0.230
Anxiety	5.64 ± 4.84	4.45 ± 3.01	−1.191	0.992, −3.164	0.238
Stress	7.91 ± 4.76	6.70 ± 3.61	−1.166	1.040, −3.289	0.248
Week **4**	14.24 ± 10.13^ac^	12.57 ± 9.83 ^d^	0.665	−3.362, 6.713	0.508
Depress	3.82 ± 4.14	3.23 ± 3.85	−0.579	−2.605, 1.435	0.565
Anxiety	4.67 ± 3.77	3.97 ± 3.17	−0.793	−2.464, 1.064	0.431
Stress	5.64 ± 3.64	5.37 ± 3.99	−0.281	−2.191, 1.651	0.780
Week 8	11.58 ± 8.19^bc^	12.63 ± 10.50^e^	−0.448	−5.779, −3.663	0.656
Depress	2.70 ± 2.94	3.60 ± 4.59	0.938	−1.022, 2.828	0.352
Anxiety	3.70 ± 3.12	3.87 ± 3.49	0.839	−1.495, 1.834	0.839
Stress	5.18 ± 3.72	5.13 ± 3.72	0.958	−1.878, 1.786	0.958

*Note:* Data expressed as a mean ± SD for continuous variables. Independent *t*‐test was used to compare between groups of continuous variables. Matching superscript pairs (aa,bb,cc,dd,ee) indicate statistically significant differences at the *p*‐value < 0.05 level, determined using repeated measures ANOVA.

Abbreviation: 95% CI, 95% confidence interval of the difference.

* Significant at the *p*‐value < 0.05.

## 4 Discussion

No significant differences were found in baseline characteristics between the two groups (Table 1). The primary aim of this study was to assess the impact of daily Spirulina softgel supplementation on sleep quality. Our findings revealed a statistically significant difference in sleep quality between the experimental and control groups at week 8, as assessed by the PSQI. Specifically, both overall sleep quality and sleep latency at week 8 were significantly better in the Spirulina group compared to the control group, with scores of 4.97 ± 1.98 and 6.73 ± 2.69, respectively (95% CI [−2.945, −0.582], *p* = 0.004). These results contradict previous clinical research on the effects of Spirulina supplementation on various health parameters, including sleep quality and mental health in patients with ulcerative colitis (Moradi et al. 2021). That study reported no statistically significant differences in overall sleep quality after 8 weeks. However, there was a difference in Spirulina dosage between the studies. In the earlier study, participants consumed 1 g/day (two 500 mg capsules/day) of Spirulina, while our study administered 2 g/day (two 1000 mg capsules/day) before bedtime. This difference in dosage may account for the varying outcomes observed in sleep quality. Additionally, the study reported a significant reduction in sleep disturbances with Spirulina supplementation (*p* = 0.03), which contrasts with our findings. Our study showed significant improvements in overall sleep quality and latency in the Spirulina group at week 8. However, no statistically significant differences in other sleep components were noted between the Spirulina and control groups. Furthermore, our study measured the SHI, revealing a significant decrease in SHI scores for both groups (lower scores indicating improved sleep hygiene), as detailed in Table 3. This suggests that participants in both groups experienced enhanced sleep hygiene, potentially contributing to improved sleep quality.

When considering the PSQI scores alongside the SHI results, it is evident that while both groups demonstrated improved sleep hygiene, only the experimental group exhibited significant improvements in sleep quality and latency at week 8 (Figure 3). This implies that daily supplementation of Spirulina at the dosage used in our study may have a more pronounced effect on these specific aspects of sleep. The sleep quality of participants receiving Spirulina gradually improved, with PSQI scores (where lower scores indicate better sleep quality) decreasing from 7.03 ± 3.52 at baseline to 5.85 ± 2.83 at week 4, and further to 4.97 ± 1.98 at week 8. In contrast, the control group initially showed improved sleep quality from baseline to week 4, with scores of 8.07 ± 3.76 and 6.40 ± 2.67, respectively. However, by week 8, the control group's sleep quality had declined to 6.73 ± 2.69 (Table 2). Additionally, this study demonstrated the placebo effect, consistent with previous research (Yeung et al. 2020; Weiss et al. 2021). The PSQI scores at week 4 indicated improvement in both groups, reflecting enhanced sleep quality. However, by the third measurement point, week 8, the Spirulina group continued to show significant improvement in sleep quality from baseline, while the control group's scores began to decline, indicating reduced sleep quality.

For mental health status, this study included participants with mild to moderately severe depression, as indicated by PHQ‐9 scores between 5 and 19. The results showed that in the Spirulina group, Depression Anxiety Stress Scales (DASS‐21) scores decreased significantly from baseline to week 4 and week 8, with scores of 19.27 ± 13.09, 14.24 ± 10.13, and 11.58 ± 8.19, respectively. Although the control group also exhibited a reduction in these scores, an increase was observed at week 8 (12.63 ± 10.50) compared to week 4 (12.57 ± 9.83) (Table 4). Similar to other clinical studies, when interventions yield tangible effects, outcomes tend to fluctuate until stabilizing at an expected level. In the control group, scores fluctuated due to the placebo effect but eventually returned to baseline levels. A previous clinical study on dietary supplementation with an extract of *Aloysia citrodora* (Lemon verbena) reported a similar trend in sleep quality (Pérez‐Piñero et al. 2024). Specifically, significant differences between the experimental and control groups were observed at the third measurement point, while the second and third points in the control group showed no significant differences.

In terms of BMI and blood pressure parameters, our study found no statistically significant differences across all three parameters over the 8 weeks. This aligns with previous literature, including a systematic review and meta‐analysis (Zarezadeh et al. 2021), which reported that Spirulina supplementation did not impact BMI over 12 weeks. Regarding blood pressure, our study similarly found no statistically significant differences between the two groups. However, prior systematic reviews and meta‐analyses (Machowiec et al. 2021) have suggested that Spirulina may have supportive effects in preventing and managing hypertension.

This study was conducted as a double‐blinded, randomized, placebo‐controlled trial to mitigate biases and confounders that could arise during the study. Furthermore, the Spirulina and placebo capsules were designed to be visually and olfactorily indistinguishable and manufactured by a certified company. Throughout the study, participants were provided with booklets to record compliance, any issues during supplementation, and adverse effects, with no severe adverse effects reported. Nonetheless, this study has certain limitations, notably the study duration. Despite a thorough literature review prior to commencement, our findings suggest that the optimal benefits might be observed with longer‐term follow‐up at 6 and 12 months. Lastly, blood draws were not performed in this study; hence, future studies should consider primary parameters such as blood samples or hormones that might clearly indicate the outcome of interest.

Previous studies have established a link between good sleep and mental health, though it remains unclear which influences the other, akin to the chicken‐and‐egg problem (Jiang et al. 2022). The findings of our study indicate that daily Spirulina supplementation can lead to significant improvements in sleep quality, sleep latency, and mental health parameters such as depression, stress, and anxiety in adults with mild to moderately severe depression. This effect is more pronounced in the experimental group than in the control group, demonstrating the potential benefits of Spirulina in managing these conditions.

## 5 Conclusion

Our study highlights two significant benefits of Spirulina supplementation: improved sleep quality and latency, and decreased depression, anxiety, and stress scores. Therefore, supplementing with Spirulina, a natural food supplement, is an option for increasing sleep quality and mental health. This is particularly beneficial for individuals with mild to moderately severe depression.

## Author Contributions

**Phenphop Phansuea:** conceptualization (equal), data curation (equal), formal analysis (equal), investigation (equal), methodology (equal), resources (equal), software (equal), supervision (equal), validation (equal), writing – original draft (equal), writing – review and editing (equal). **Kittipat Chotchindakun:** project administration (equal), resources (equal), validation (equal). **Yuraporn Sahasakul:** investigation (equal), project administration (equal), writing – review and editing (equal). **Karunpong Phattaramarut:** supervision (equal), validation (equal), visualization (equal). **Panwong Kuntanawat:** conceptualization (equal), funding acquisition (equal), methodology (equal), resources (equal), supervision (equal), validation (equal), visualization (equal), writing – review and editing (equal).

## Ethics Statement

This study was conducted in accordance with the Declaration of Helsinki and approved by the Mahidol University Central Institutional Review Board (COA No. MU‐CIRB 2023/184.1312; Date of Approval: December 13, 2023).

## Consent

All participants were informed of the purpose and details of this study, and their participation was entirely voluntary. Participants provided consent via a screening question. Participants who did not agree to participate were excluded. Informed consent was obtained from all participants involved in the study.

## Conflicts of Interest

The authors declare no conflicts of interest.

## Supporting information


Data S1.

## Data Availability

Data associated with the paper is available upon request.
