# Supplementary material for: Effectiveness of an Arthrospira platensis (Spirulina) Softgel Supplementation on Sleep Quality, Mental Health Status, and Body Mass Index in Mild to Moderately Severe Depression Adults: A Double‐Blinded, Randomized, Placebo‐Controlled Trial
Source: Food Sci Nutr. 2025 Mar 5;13(3):e70082. doi: 10.1002/fsn3.70082 (PMC11882752; doi:10.1002/fsn3.70082)
Supplement: Supplementary file 1 — Data S1. [file FSN3-13-e70082-s001.docx]

Appendix

Supplementary A

Appointment plan

**Initial Appointment**

**Estimated Duration:** Approximately 1.5 hours

The research team began by providing a comprehensive explanation of the project to the participants, who then acknowledged their understanding and signed the participation documents. Preliminary data were recorded through individual interviews utilizing the General Interview Questionnaires (GIQ) consisting of 14 items, followed by blood pressure monitoring using Omron HEM-1040, height measurement, and body composition analysis conducted using the INBODY 270 apparatus by the research team at the Institute of Nutrition, Mahidol University. This phase took approximately 30 minutes to 1 hour per participant. Subsequently, participants proceeded with a self-administered questionnaire comprising 43 items, which took approximately 20-30 minutes. The questionnaire included:

1. Depression Anxiety Stress Scales (DASS-21) consisting of 21 items.
2. The Pittsburgh Sleep Quality Index (PSQI) consisting of 9 items.
3. Sleep Hygiene Index (SHI) consisting of 13 items.

Upon completion of the questionnaire phase, each participant was assigned a random number (ranging from 01-66) by the research team, who utilized a randomization table to select the number. Simultaneously, the research team prepared and dispensed experimental or control packages corresponding to the assigned code. Each package was designated for a duration of 4 weeks and contained four sub-packets, each containing 14 softgel capsules, resulting in a total of 56 softgel capsules for each participant. The large packet was labeled with the code and week, for example, Code 01 Packet 1 for Week 1, Code 01 Packet 2 for Week 2, and so forth. Participants were instructed to consume 2 softgel capsules before dinner daily and to record their daily consumption of the product, as well as any abnormal or undesirable effects experienced. It was important to note that the research personnel responsible for distributing the products remained unaware of whether the packet contained an experimental or controlled substance. This process took approximately 10 minutes.

**Second Appointment**

**Estimated Duration:** Approximately 1 hour

Upon the completion of four weeks, participants attended the research appointment as scheduled. Upon arrival, the research team collected any unused softgel capsules and requested the return of daily product intake records, along with documentation of any abnormal or undesirable effects experienced. Subsequently, participants underwent blood pressure monitoring using Omron HEM-1040, a body composition analysis using the INBODY 270 apparatus, administered by the research team. This phase took approximately 30 minutes per participant. Following this, participants proceeded with a self-administered questionnaire comprising 43 items, which took approximately 15-20 minutes. The questionnaire included:

1. Depression Anxiety Stress Scales (DASS-21) consisting of 21 items.
2. The Pittsburgh Sleep Quality Index (PSQI) consisting of 9 items.
3. Sleep Hygiene Index (SHI) consisting of 13 items.

Upon completion of the questionnaire phase, each participant was assigned the same code as in the previous appointment (ranging from 01-66) by the research team. Simultaneously, the research team prepared and dispensed experimental or control packages for another four-week duration, as previously explained. Participants were also instructed on consumption and the return of any unused softgel capsules. The research team provided compensation and covered travel expenses for the participants. Additionally, participants were scheduled for their next appointment, specifying the date and time. This process took approximately 10 minutes.

**Third Appointment**

**Estimated Duration:** Approximately 1 hour

Following an additional four-week interval, participants attended the research appointment as scheduled. Upon arrival, the research team collected any unused softgel capsules and requested the return of daily product intake records, along with documentation of any abnormal or undesirable effects experienced. Subsequently, participants underwent blood pressure monitoring using Omron HEM-1040, a body composition analysis using the INBODY 270 apparatus, administered by the research team. This phase took approximately 30 minutes per participant. Following this, participants proceeded with a self-administered questionnaire comprising 43 items, which took approximately 15-20 minutes. The questionnaire included:

1. Depression Anxiety Stress Scales (DASS-21) consisting of 21 items.
2. The Pittsburgh Sleep Quality Index (PSQI) consisting of 9 items.
3. Sleep Hygiene Index (SHI) consisting of 13 items.

Upon completion of the questionnaire phase, the research team provided compensation and covered travel expenses for the participants. It is noteworthy that the selected daily dosage of 2 grams aligned with the previous study, where no reports of adverse effects or undesired events were noted among the 12 participants in the intervention group (receiving 6 grams/day of Spirulina supplementation). Thus, this study set the dosage at 2 grams/day, three times less than the previous study. In this study, participants were instructed to consume either the Spirulina supplement “Sup Blue” (2 grams/day) or a placebo (2 grams of syrup) for a duration of 8 weeks.

The assessment of sleep quality and mental health status was conducted using a combination of tools, including the DASS-21, Thai-PSQI, and SHI. Additionally, BMI measurements were taken in the 4th week and at the conclusion of the study in the 8th week. In the event that the study yielded results in line with the stated objectives, the research team would proceed to contact and provide the product to the placebo group for the subsequent phase of the study.

Supplementary B


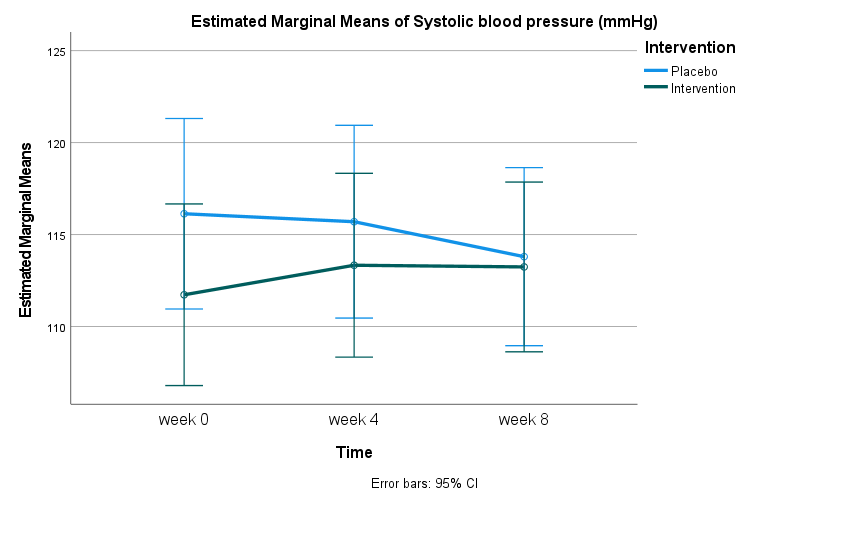


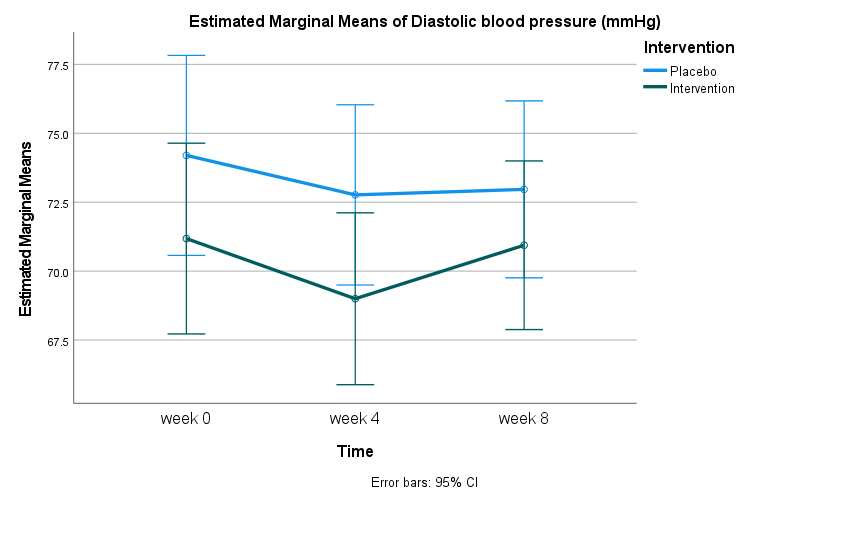


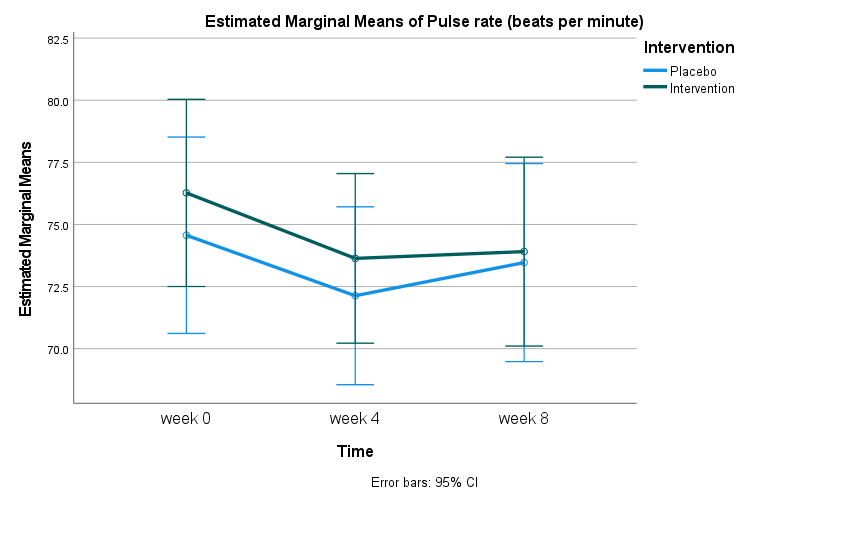


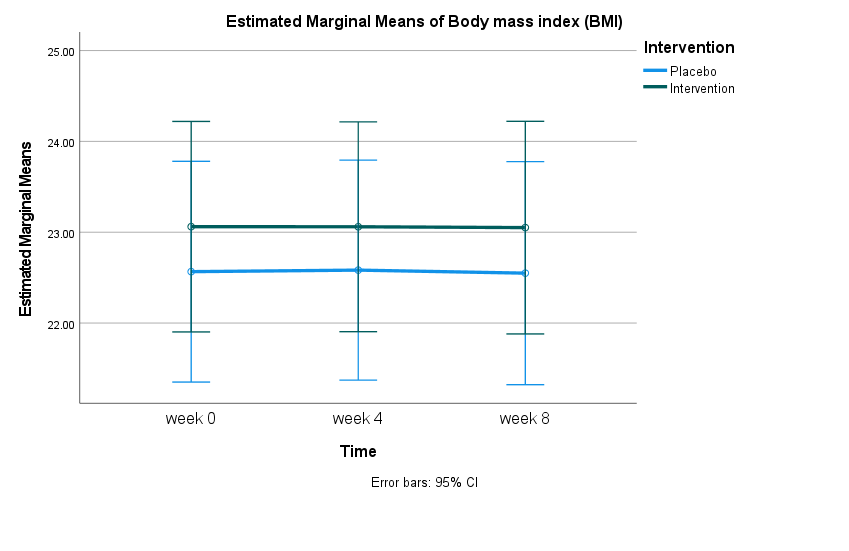


**Supplementary C****Comparison of Pittsburgh Sleep Quality Index (PSQI) Scores in the experimental group at baseline, week 4, and week 8 using Repeated Measures ANOVA**

|  | **Sum of Squares** | **df** | **Mean Square** | **F** | ***p* value** | **Partial Eta Squared** |
| --- | --- | --- | --- | --- | --- | --- |
| Between groups | | | | | | |
| Contrast | 19.613 | 1 | 19.613 | 3.032 | .087 | .047 |
| Error | 394.560 | 61 | 6.468 |  |  |  |
| Within groups | | | | | | |
| Time | 104.396 | 1.491 | 70.010 | 15.111 | <.001* | .199 |
| Time * Intervention | 11.698 | 1.491 | 7.845 | 1.693 | .196 | .027 |
| Error (Time) | 421.434 | 90.961 | 4.633 |  |  |  |
| * Significant at the p-value < 0.05, Greenhouse-Geisser was conducted. | | | | | | |

**Supplementary D**

**Comparison of Sleep Hygiene Index (SHI) scores in the experimental group at baseline, week 4, and week 8 using Repeated Measures ANOVA**

|  | **Sum of Squares** | **df** | **Mean Square** | **F** | ***p* value** | **Partial Eta Squared** |
| --- | --- | --- | --- | --- | --- | --- |
| Between groups | | | | | | |
| Contrast | 21.836 | 1 | 21.836 | .462 | .499 | .008 |
| Error | 2881.815 | 61 | 47.243 |  |  |  |
| Within groups | | | | | | |
| Time | 658.570 | 2 | 329.285 | 21.270 | <.001* | .259 |
| Time * Intervention | 32.707 | 2 | 16.354 | 1.056 | .351 | .017 |
| Error (Time) | 1888.721 | 122 | 15.481 |  |  |  |
| * Significant at the p-value < 0.05, Sphericity assumed was conducted. | | | | | | |

**Supplementary E**

**Comparison of Depression Anxiety Stress Scales (DASS-21) scores in the experimental group at baseline, week 4, and week 8 using Repeated Measures ANOVA**

|  | **Sum of Squares** | **df** | **Mean Square** | **F** | ***p* value** | **Partial Eta Squared** |
| --- | --- | --- | --- | --- | --- | --- |
| Between groups | | | | | | |
| Contrast | 34.694 | 1 | 34.694 | .400 | .530 | .007 |
| Error | 5293.077 | 61 | 86.772 |  |  |  |
| Within groups | | | | | | |
| Time | 939.235 | 1.705 | 550.918 | 16.515 | <.001* | .213 |
| Time * Intervention | 189.267 | 1.705 | 111.016 | 3.328 | .045* | .052 |
| Error (Time) | 3469.135 | 103.996 | 33.358 |  |  |  |
| * Significant at the p-value < 0.05, Greenhouse-Geisser was conducted. | | | | | | |
